# Distinct Contributions of Different Domains within the HIV-1 Gag Polyprotein to Specific and Nonspecific Interactions with RNA

**DOI:** 10.3390/v12040394

**Published:** 2020-04-02

**Authors:** Tomas Kroupa, Siddhartha A. K. Datta, Alan Rein

**Affiliations:** HIV Dynamics and Replication Program, Center for Cancer Research, National Cancer Institute, Frederick, MD 21702, USA; tomas.kroupa@nih.gov (T.K.); dattasi@mail.nih.gov (S.A.K.D.)

**Keywords:** HIV, Gag, retroviral, packaging, retroviral assembly

## Abstract

Viral genomic RNA is packaged into virions with high specificity and selectivity. However, in vitro the Gag specificity towards viral RNA is obscured when measured in buffers containing physiological salt. Interestingly, when the binding is challenged by increased salt concentration, the addition of competing RNAs, or introducing mutations to Gag protein, the specificity towards viral RNA becomes detectable. The objective of this work was to examine the contributions of the individual HIV-1 Gag polyprotein domains to nonspecific and specific RNA binding and stability of the initial protein-RNA complexes. Using a panel of Gag proteins with mutations disabling different Gag-Gag or Gag-RNA interfaces, we investigated the distinct contributions of individual domains which distinguish the binding to viral and nonviral RNA by measuring the binding of the proteins to RNAs. We measured the binding affinity in near-physiological salt concentration, and then challenged the binding by increasing the ionic strength to suppress the electrostatic interactions and reveal the contribution of specific Gag–RNA and Gag–Gag interactions. Surprisingly, we observed that Gag dimerization and the highly basic region in the matrix domain contribute significantly to the specificity of viral RNA binding.

## 1. Introduction

The retroviral genomic RNA typically represents less than 1% of the total RNA in the infected cell but it is specifically packaged into new virions. The region of the viral RNA responsible for directed packaging into newly formed particles is called the packaging signal (Ψ). In HIV-1 it is an ~80 to 150 nucleotide segment located within the 5′ UTR of the viral genome [1,2]. In the absence of Ψ-containing RNA, virtually any cytoplasmic mRNA can be packaged into the virus particle in vivo [3]. In vitro, as far as is known, any nucleic acid > ~30 bases long can support assembly [4]. However, when Ψ-containing RNA is present in the cell, it is preferentially packaged with high specificity (in cell culture >90% of the virus particles contain viral RNA) [5].

The main partner for the viral RNA in the packaging process is the retroviral protein Gag. HIV-1 Gag is a ~55 kDa polyprotein composed of several independently folded domains connected by flexible linkers. From the N- to C-terminus these domains are matrix (MA), capsid (CA), spacer peptide 1 (SP1), nucleocapsid (NC), spacer peptide 2 (SP2), and p6 [6]. The main Gag domain responsible for RNA binding is NC, which has two zinc fingers and positively charged amino acids near its N-terminus and between the fingers. Although the main role of the MA domain is targeting of Gag polyprotein to the plasma membrane and the interaction with cellular membranes (reviewed in [7]), it also contributes to RNA binding through its highly basic region (HBR), a patch of basic residues spanning amino acids 15 to 32 of MA [8,9,10].

In vitro Gag-RNA binding experiments showed that in physiological salt concentrations, the affinity of Gag for Ψ and non-Ψ RNA is comparable. However, when binding is measured under conditions of higher salt concentrations which suppress nonspecific electrostatic interactions, preferential binding to Ψ RNA is revealed [11,12]. It has also been shown that mutations in MA, CA, and NC domains attenuate the Gag binding to Ψ RNA [11].

A previous SHAPE structure-probing analysis of the HIV-1 5′ UTR revealed that the main NC interaction domain within Ψ consists of seven short stretches containing unpaired guanosines that are recognized by the NC zinc fingers [13]. It has been shown that mutating these unpaired guanosines interfered with the packaging of the RNA in virus-producing cells [14] and led to the less efficient assembly of the viral particles in vitro [15].

However, the nature of the difference between Gag’s interaction with Ψ RNAs and non-Ψ RNAs is still unclear. Especially, how is it possible that the viral RNA is packaged into the newly formed virus almost exclusively? We have reported that Gag protein assembles much more efficiently upon interaction with the viral genomic RNA [15] than with control RNAs, and suggested that this is the explanation for selective packaging of genomic RNA in vivo, where it is surrounded by a great excess of other RNAs [1]. The mechanism of this selective packaging is still not well understood.

In this work, we analyzed which Gag domains and multimerization interfaces are important for binding to Ψ RNA and non-Ψ RNA. These experiments relied on microscale thermophoresis (MST) [16,17], which, up to now, has not been widely applied in this area. MST is a solution-state technique that follows the movement of fluorescent molecules under microscopic thermal gradients. Since the mobility is influenced by size, charge, and hydration shell of binding partners, it offers a very sensitive method to monitor binding reactions [16,17]. We present the binding data from MST measurement of nine selected Gag protein mutants with mutations in MA, CA, or NC disabling different Gag–Gag or Gag–RNA interaction interfaces, and we compare them with the binding of wild-type (WT) Δp6 Gag. It should be noted that the “Gag” protein used in these experiments differs in two ways from the authentic Gag protein expressed in infected mammalian cells. First, it does not have the fatty-acid myristoyl modification at its extreme N-terminus, and second, it does not contain the p6 domain. We cannot exclude the possibility that p6 plays a role in interactions with RNA, as has been suggested recently [18,19].

## 2. Materials and Methods 

### 2.1. RNA Constructs and Preparation

RNAs were produced by in vitro transcription of linearized plasmids containing the T7 promoter, as previously described [11]. Ψ RNA is 400 nucleotides long, constructed from HIV-1 (strain NL4-3) 5′UTR (nucleotides 201 to 600). We used the reverse complement of this RNA as a non-Ψ, control RNA. The RNAs were Cy5-labeled by ligating pCp-Cy5 (Jena Bioscience GmbH, Jena, Germany) to the 3′-end of the RNA with T4 RNA ligase (New England Biolabs, Ipswich, MA, USA) [20]. Prior to the measurements both RNAs were incubated to permit dimerization, as previously described [11,21] and diluted with measuring buffer so that the final RNA concentration in each sample was 5 nM. Under these conditions, Ψ RNA forms dimers while the reverse complement RNA does not, as determined by native agarose gel electrophoresis. The proteins and RNA were mixed and incubated overnight at 4 °C prior to the measurements.

### 2.2. Gag Protein Mutants

All the proteins were non-myristoylated and derived from WT Δp6 Gag (isolate BH10), expressed without any affinity tags in BL21(De3)pLysS *E. coli* and purified following a previously published protocol [22], followed by an additional size exclusion chromatography purification step with Superose 12 10/300GL (GE Healthcare Life Sciences, Marlborough, MA, USA) and ion-exchange chromatography with a Bio-Scale Mini Macro-Prep High S (Bio-Rad Laboratories, Hercules, CA, USA). We estimate, based on SDS-PAGE and Coomassie blue staining, that each protein preparation was at least 85% pure.

8N Gag [23] has eight basic residues (R15, K18, R20, R22, K26, K27, K28, and K30) in the N-terminal region of the MA domain replaced with asparagines. HBR switch Gag has arginine residues (15, 20, and 22) mutated to lysines and lysines (18, 26, 27, 28, and 30) mutated to arginines. ΔMA Gag [24] is lacking amino acids 16 to 99 in the MA domain. 

WM Gag [25] has tryptophan W316 and methionine M317 replaced by alanine, preventing Gag dimerization in solution [25]. Δ1 SP1 Gag has 1 amino acid deletion (ΔA364) at the very N-terminus of SP1, preventing the formation of the six-helix bundle, a significant interaction interface of the hexamers in the viral lattice [26,27].

SSHC Gag [11] has the first two cysteines in each NC zinc finger (C392, C395, C413, and C416) replaced by serines, destroying the protein’s ability to coordinate zinc ions. NC 4A Gag (also known as 310 Gag [11]) has basic amino acid residues R406, R409, K410, and K411 between the two zinc fingers in the NC domain mutated to alanines. FW Gag [28] has residues F393 and W414 in the NC domain mutated to alanines. These two aromatic residues have been shown to make hydrophobic contacts and hydrogen bonding with RNA [29]. NC 10A Gag has 10 basic amino acids in the NC domain (residues K391, K397, R403, R406, R409, K410, K411, K415, K418, and K424) replaced by alanines, neutralizing the protein charge and weakening the electrostatic RNA binding.

### 2.3. Thermophoresis Measurements

Thermophoresis [16] measurements were performed in premium coated capillaries on a Monolith NT.115 instrument (Nanotemper Technologies GmbH, Munich, Germany). Samples were incubated 20 min at 22 °C after loading into measuring capillaries. All experimental measurements were performed with temperature control set to 22 °C. Infrared laser power was 20% for all measurements. At least 3 experimental replicates were performed in all cases. Measuring buffer composition was 100 mM or 300 mM NaCl, 30 mM phosphate (pH 7.5), 0.05% Tween20, 0.1 mM phenylmethylsulfonyl fluoride (PMSF), and 1 mM β-mercaptoethanol (βME).

### 2.4. Fitting the Experimental Data and Statistics

All fitting of experimental data was performed using R (version 3.6.1) with installed mixtox package and binding equation:

*F* = *F_min_* + (*F_max_* − *F_min_*)/(1 + (*EC*50/*conc*)*^hill^*), (1)

where *F_min_* is unbound response, *F_max_* is bound response, *hill* is Hill coefficient, and *conc* is concentration of the protein.

The statistical significance of the differences between proteins binding to Ψ and to reverse complement RNAs was evaluated using two-tailed t-test with significance level 0.05. In 100 mM NaCl, the differences in binding were not statistically significant. In 300 mM NaCl, the differences in binding of 8N, HBR switch, and WM Gag were not significant. Proteins for which EC50 values could only be estimated, i.e., FW, NC 4A, NC 10A, and SSHC Gag were not evaluated for statistical significance. The differences in binding of WT, ΔMA, and Δ1 SP1 Gag in 300 mM NaCl were significant with *p* < 0.05.

## 3. Results

Out of the six domains that comprise full-length Gag polyprotein, four of them are directly involved in Gag–Gag and/or Gag–RNA interactions during the virus particle assembly. They are MA, CA, SP1, and NC domains (reviewed in [30]). The NC domain is responsible mainly for RNA binding and RNA chaperone activity; the CA domain for Gag assembly; the SP1 linker forms a six-helix bundle that stabilizes the immature viral lattice; and the MA domain is responsible for membrane binding, Gag targeting to the plasma membrane, and Env incorporation. It has become clear in recent years that in addition to these activities, MA can also bind RNAs [8,31].

To address the question of which parts of the Gag polyprotein are important for selective packaging of viral RNA we prepared nine mutants of Gag that impair different regions known to participate in Gag–RNA or Gag–Gag interactions. The mutations can be divided into three groups. The first group has mutations in the MA domain. It is either a large deletion in MA (ΔMA Gag) or mutations altering the HBR of MA (8N Gag and HBR switch Gag). The second group contains mutants that have impaired the ability to form Gag dimers (WM Gag) or the six-helix bindle (Δ1 SP1 Gag). The last group has mutations in the NC domain affecting the zinc fingers (SSHC Gag), the charge of the NC domain (NC 4A Gag and NC 10A Gag), or the aromatic residues that contribute to RNA binding (FW Gag). 

**Figure 1:**
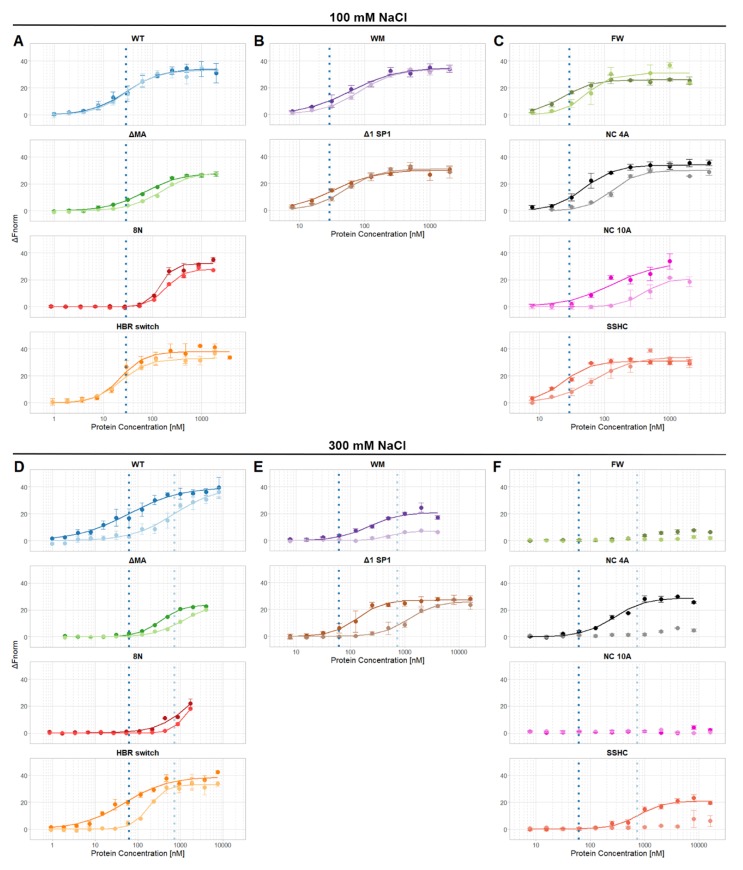
Binding curves of individual proteins. Panels (**A**–**C**) are binding curves obtained in buffer containing 100 mM NaCl; panels (**D**–**F**) are binding curves obtained in buffer containing 300 mM NaCl. Dashed vertical lines represent EC50 values of WT Gag with Ψ (dark blue) and reverse complement (light blue) RNAs. (**A**,**D**) WT and matrix mutants, (**B**,**E**) multimerization mutants, (**C**,**F**) nucleocapsid mutants. The p6 domain is not present in any of these proteins.

**Figure 2:**
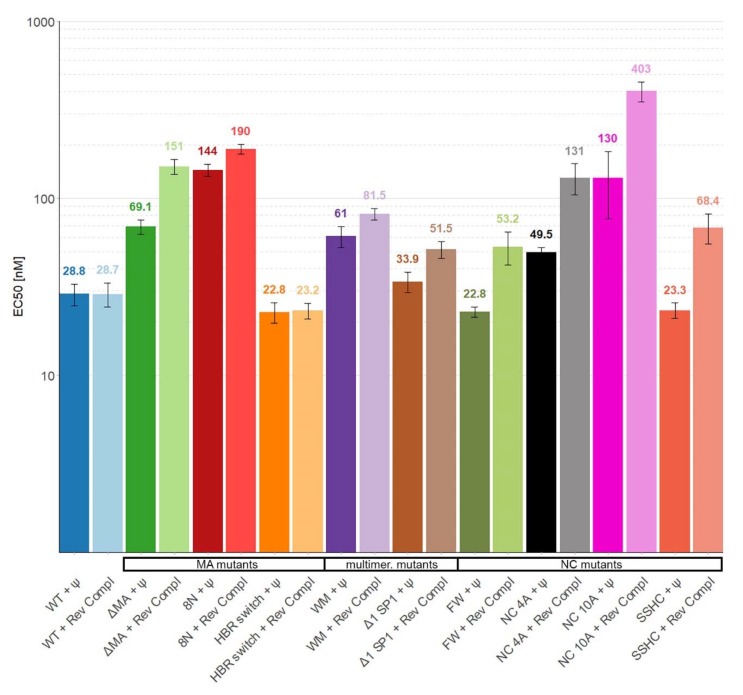
The affinity of WT Gag Δp6 and mutants for Ψ and reverse complement RNAs in buffer containing near-physiological salt (100 mM). None of the differences between binding to Ψ and reverse complement RNAs for individual proteins is statistically significant. Error bars represent 95% confidence interval of EC50 values.

**Figure 3:**
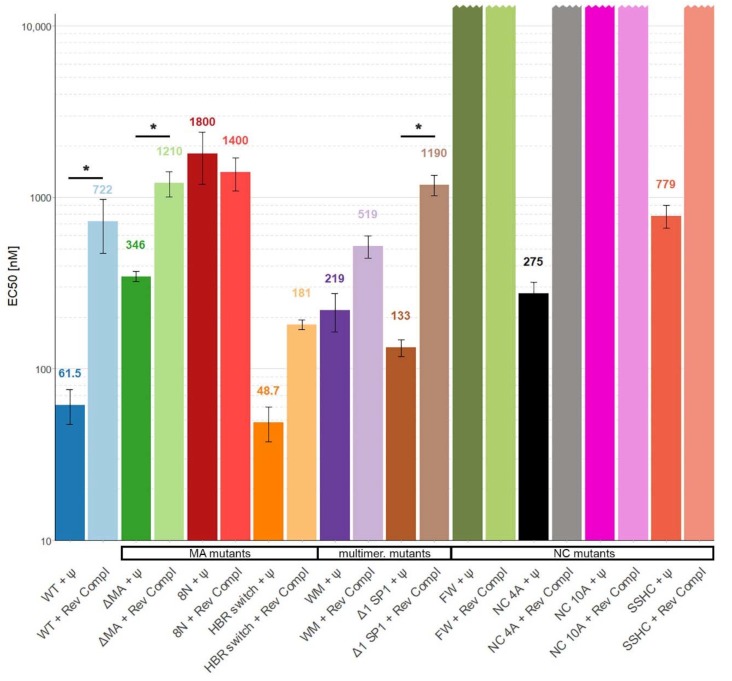
The affinity of WT Gag Δp6 and mutants to Ψ and reverse complement RNAs in buffer with 300 mM NaCl. *, *p* < 0.05. Error bars represent 95% confidence interval of EC50 values.

The binding of the proteins to Ψ and reverse complement RNA was then tested in buffers containing near-physiological salt (100 mM NaCl) and at a higher salt concentration (300 mM); the resulting curves are shown in Figure 1. Then, the midpoints of the binding curves (“EC50′s”) were extracted and are shown in Figure 2 (100 mM NaCl) and Figure 3 (300 mM NaCl). The findings in 100 mM NaCl confirmed previous findings [11,12] that under such conditions the binding affinities of WT Gag to Ψ and reverse complement RNAs are comparable (Figure 1 and Figure 2). Different EC50 values were obtained with those mutants that affect the overall charge of the Gag polyprotein. Specifically, 8N Gag and NC10A Gag showed reduced binding to both Ψ and control RNAs. This result confirms that in near-physiological salt concentrations the main driving force of the Gag-RNA binding is nonspecific electrostatic interactions [11,12].

Therefore, we increased the salt concentration in the measuring buffer to 300 mM to reduce the nonspecific electrostatic interactions. Under these conditions, we were able to detect approximately 12 times stronger binding of WT Gag to Ψ RNA than to reverse complement RNA (Figure 3). 

If the basic amino acids in the HBR in the MA domain were mutated to asparagine (8N Gag) the affinity of Gag to RNA was decreased more than 25-fold and there was no selectivity towards Ψ RNA. A similar but weaker effect was observed with ΔMA Gag, which bound Ψ RNA approximately 3.5 times stronger than reverse complement RNA (Figure 1D and Figure 3). Surprisingly, the HBR switch Gag and WT Gag binding affinities towards Ψ RNA were similar but the affinity towards reverse complement RNA was approximately four times higher for the HBR switch Gag. These results with the MA mutants suggest that HBR plays a role in selective binding of Ψ RNA and that the selectivity is not driven solely by the positive charge of HBR because switching arginines and lysines (preserving the overall charge of the mutant) led to a stronger binding of reverse complement RNA.

Destroying the ability to form the six-helix bundle (Δ1 SP1 Gag) in Gag mildly affected the binding to RNA (two-fold difference in binding as compared with WT Gag). Interestingly, preventing Gag from forming dimers (WM Gag) decreased the affinity towards Ψ RNA 3.5 times but the affinity towards reverse complement RNA did not change significantly (Figure 1E and Figure 3). This suggests that dimerization contributes to the specific binding of Ψ RNA.

We were not able to detect any binding of NC 10A Gag and FW Gag with either of the RNAs and no binding of SSHC Gag and NC 4A Gag with reverse complement RNAs (Figure 1F and Figure 3) in 0.3M NaCl, up to the maximum protein concentration used (15 µM for NC 10A, FW, and SSHC Gag and 7.5 µM in the case of NC 4A Gag). Therefore, we estimate that the EC50 values in 300 mM NaCl for NC 10A, FW, and SSHC Gag proteins are >30 µM and >15 µM for NC 4A Gag.

Destroying the ability of Gag to coordinate zinc ions (SSHC Gag) led to an approximately 12-fold decrease in the binding affinity towards Ψ RNA and no detectable binding of reverse complement RNA. This result suggests that zinc fingers, while important, are not entirely responsible for the preferential binding of Ψ RNA. The lack of binding of NC 4A Gag to the reverse complement RNA, while partially preserving the ability to bind Ψ RNA, confirms the hypothesis that NC 4A Gag binds to Ψ RNA through non-electrostatic interactions that ensure the preferential binding of viral RNA [11]. The absence of any detectable binding of the FW Gag and NC 10A Gag in 300 mM NaCl proves the high importance of these residues for RNA binding. Interestingly, in 100 mM NaCl, the binding of FW Gag to Ψ RNA was not impacted.

In summary, the presented results demonstrate the importance of MA’s HBR for RNA binding and the supportive effect of Gag dimerization in selective binding of Ψ RNA. Although more experiments need to be performed to truly understand the underlying mechanism, these findings show the need to study Gag-RNA binding using constructs containing the MA domain and offer new evidence about the role of individual Gag domains in the initial stages of viral particle assembly.

## 4. Discussion

We present in vitro binding experiments of multiple HIV-1 Gag polyprotein mutants, with lesions affecting various Gag–Gag or Gag–RNA interaction interfaces, with RNA containing the Ψ sequence or with a control RNA. The results presented here show that the positively charged and aromatic residues in the NC domain are crucial for any RNA binding to occur. Strikingly, if these are present, then, the positively charged amino acids in the HBR of MA serve as an additional Gag-RNA binding interface that also contributes to the specific binding to Ψ RNA. The formation of the six-helix bundle and especially Gag dimerization contribute to tighter RNA binding even below the concentration needed for Gag polyprotein to dimerize in solution. This suggests that specific Gag–Gag interactions could be generated in the nascent Ψ RNA-Gag complexes.

Taken together, the data help to identify the regions of Gag polyprotein that contribute to specific and nonspecific RNA binding. Our data confirmed that the most important determinants for Gag binding to RNA are the basic amino acid residues within the NC domain and the two aromatic amino acid residues in the NC zinc fingers. In high salt buffer, we were not able to detect any binding of FW Gag and NC 10A Gag to any RNA. On the basis of the highest protein concentration used in the experiments we estimated the EC50 values to be >30 µM. Therefore, we conclude that these two features are crucial for RNA binding.

When the basic and aromatic residues were present, we observed binding to Ψ RNA even in the absence of zinc fingers (SSHC Gag) or with four basic amino acids between the zinc fingers neutralized (NC 4A Gag). The SSHC results are in accordance with previously published experiments [11,12]. The damage to RNA binding is in harmony with in vivo RNA packaging data published by Poon et al. [32], which showed that both of these mutants packaged significantly less genomic RNA than wild-type Gag. Interestingly, even those mutant particles containing genomic RNA were apparently non-infectious, suggesting that the mutated residues perform other essential functions in replication, in addition to their role in RNA packaging. Other in vivo analyses of mutants affecting the zinc fingers are also in agreement with these conclusions [33].

It is striking that the multimerization mutants (WM and Δ1 SP1 Gag) affect binding to RNA because binding was measured at a protein concentration in which free WT Gag is monomeric. The K_d_ for Gag dimerization is 5.5 µM [25]. Somewhat analogous observations were also published by Zhao et al. [34], who found that Gag dimerization on a very short oligonucleotide was diminished by the WM mutation, although the experiments were performed well below the K_d_ for Gag–Gag interaction in free solution. In fact, since the K_d_ for Gag–RNA interactions is in the nanomolar realm while Gag–Gag interactions are in the micromolar realm, it is clear that binding nearby sites on an RNA molecule could lead to Gag–Gag interactions at far lower concentrations than can occur in solution. This could help explain how binding to RNA promotes particle assembly. 

The results of our binding experiments slightly differ from those published by Comas-Garcia et al. [11] which were performed with shorter fluorescently labeled RNA constructs (175 nt) by monitoring fluorescence quenching. In that experimental setup it was not possible to detect any binding of WM, NC 4A, and SSHC Gag to non-Ψ RNA in buffers with NaCl concentration above 200 mM. There were also no detected differences from WT protein in the binding of 8N Gag and WM Gag to dimeric Ψ RNA at 300 mM NaCl. We believe that the difference could be caused by the use of shorter RNA constructs and a different measuring buffer; a direct comparison of the fluorescence quenching and microscale thermophoresis binding experiments with a 400 nt long Ψ RNA construct by Comas-Garcia et al. [15] found negligible differences between the two experimental techniques. It is also possible that thermophoresis is inherently more sensitive, with regard to weak binding, than the quenching assay.

The role of the MA domain in interactions of Gag with RNA is complex. It has been reported [35] that a Gag protein lacking the entire domain can support virus replication, indicating that MA is unnecessary for selective packaging in vivo. Although it has been known that the MA domain binds RNA (reviewed in [31]) our results suggest that its role in the recognition of viral RNA is more important than previously thought. Neutralizing the basic charge of the HBR (8N Gag) or deleting a large portion of the MA domain (ΔMA Gag) led to a significant decrease in binding affinity and the loss of specificity towards Ψ RNA. Additionally, swapping arginines and lysines in the HBR raised the affinity of Gag for reverse complement RNA ~four-fold. Similarly, Todd et al. [36] in their work studying Gag-liposome binding observed that the binding of HBR switch Gag (a slightly different mutant from this work, as we changed R15 to K and they did not) was affected more than the binding of WT Gag by the presence of tRNA^Lys3^. It has been also reported that mutations in the HBR change Gag localization in cells in vivo [37]. Our results show that HBR in the MA domain also makes a major contribution to Gag-RNA binding. However, this binding is dependent on the presence of positively charged and aromatic amino acids in the NC domain. This dependence suggests that NC provides the initial interaction energy, but that this interaction is enhanced by further electrostatic interaction with the positive amino acids within the MA domain. The experiments of Todd et al. [36] led to a similar conclusion. The explanation of this observation could be in the three-dimensional (3D) structure of Ψ RNA that could provide a suitable scaffold for Gag molecules to bind by both the NC and MA domains, leading to more efficient particle assembly. However, more experimental data is needed to test this hypothesis.
